# Prioritizing zoonotic diseases in Ethiopia using a one health approach

**DOI:** 10.1016/j.onehlt.2016.09.001

**Published:** 2016-09-15

**Authors:** Emily G. Pieracci, Aron J. Hall, Radhika Gharpure, Abraham Haile, Elias Walelign, Asefa Deressa, Getahun Bahiru, Meron Kibebe, Henry Walke, Ermias Belay

**Affiliations:** a National Center for Emerging and Zoonotic Infectious Diseases, Centers for Disease Control and Prevention, Atlanta, GA, USA; b Epidemic Intelligence Service, Center for Surveillance, Epidemiology and Laboratory Services, Centers for Disease Control and Prevention, Atlanta, GA, USA; c National Center for Immunization and Respiratory Diseases, Centers for Disease Control and Prevention, Centers for Disease Control and Prevention, Atlanta, GA, USA; d Ethiopian Public Health Institute, Addis Ababa, Ethiopia; e Ethiopian Ministry of Livestock and Fishery Resources, Addis Ababa, Ethiopia; f Ethiopian Ministry of Environment and Forestry, Addis Ababa, Ethiopia

**Keywords:** One health, Zoonotic disease, Disease prioritization, Ethiopia

## Abstract

**Background:**

Ethiopia has the second largest human population in Africa and the largest livestock population on the continent. About 80% of Ethiopians are dependent on agriculture and have direct contact with livestock or other domestic animals. As a result, the country is vulnerable to the spread of zoonotic diseases. As the first step of the country's engagement in the Global Health Security Agenda, a zoonotic disease prioritization workshop was held to identify significant zoonotic diseases of mutual concern for animal and human health agencies.

**Methods:**

A semi-quantitative tool developed by the US CDC was used for prioritization of zoonotic diseases. Workshop participants representing human, animal, and environmental health ministries were selected as core decision-making participants. Over 300 articles describing the zoonotic diseases considered at the workshop were reviewed for disease specific information on prevalence, morbidity, mortality, and DALYs for Ethiopia or the East Africa region.

Committee members individually ranked the importance of each criterion to generate a final group weight for each criterion.

**Results:**

Forty-three zoonotic diseases were evaluated. Criteria selected in order of importance were: 1)severity of disease in humans, 2)proportion of human disease attributed to animal exposure, 3)burden of animal disease, 4)availability of interventions, and 5)existing inter-sectoral collaboration. Based on the results from the decision tree analysis and subsequent discussion, participants identified the following five priority zoonotic diseases: rabies, anthrax, brucellosis, leptospirosis, and echinococcosis.

**Discussion:**

Multi-sectoral collaborations strengthen disease surveillance system development in humans and animals, enhance laboratory capacity, and support implementation of prevention and control strategies. To facilitate this, the creation of a One Health-focused Zoonotic Disease Unit is recommended. Enhancement of public health and veterinary laboratories, joint outbreak and surveillance activities, and intersectoral linkages created to tackle the prioritized zoonotic diseases will undoubtedly prepare the country to effectively address newly emerging zoonotic diseases.

## 1 Introduction

Most known human infectious diseases and approximately three-quarters of newly emerging infections come from animals [1], [2]. Zoonotic diseases have the potential to impact society in three main ways: (1) they threaten the health of animals resulting in illness, loss of productivity, and death; (2) they threaten the livelihood of people dependent on livestock as a major source of income; and (3) they cause illness and death in people, which in turn causes additional economic and societal loss.

Ethiopia has the second largest human population in Africa and the largest livestock population on the continent [3], [4], [5], [6]. Ethiopia is particularly vulnerable to the effect of zoonotic diseases because the economy is largely dependent on agriculture [7], [8] and roughly 80% of households have direct contact with domestic animals, creating an opportunity for infection and spread of disease [2], [9]. Ethiopia also ranks very high in the health burden of zoonotic diseases and in having a large population of poor livestock keepers [10]. Meanwhile, the lack of coordination among human and animal health sectors coupled with inadequate resources for public health systems have been prominent factors that have contributed to weak surveillance systems and less efficient and ineffective response to public health threats in the country. Therefore, having a mutually agreed and prioritized agenda among key sectors is crucial for resource allocation and strengthening zoonotic disease surveillance systems in the country.

Ethiopia's Growth and Transformation Plan, developed under the guidance of the United Nations, intends to further increase the livestock population and maximize their productivity. This requires a parallel national strategy to prevent and control the most significant zoonotic diseases, which is also a component of the Global Health Securities Agenda (GHSA). GHSA is an initiative developed by the US government with other international collaborators to address the gaps that exist in many countries in meeting the International Health Regulations and the Performance of Veterinary Services (PVS) pathway. GHSA has three strategies — Predict, Respond, and Prevent — and eleven packages were developed to achieve the strategies [11]. One of these action packages is addressing the burden of zoonotic diseases. Because a large number of zoonotic diseases endemically occur in Ethiopia, a prioritization process was necessary to identify the most critical zoonotic diseases that should be jointly addressed by animal and human health agencies to maximize impact on the health of people and animals in Ethiopia. The present article describes the first semi-quantitative, multi-sectoral process used for prioritization of zoonotic diseases in Ethiopia.

## 2 Methods

The prioritization process involved a semi-quantitative tool developed at the U.S. Centers for Disease Control and Prevention (CDC). The methods have been described in detail by Rist et al. [12]. An in-country workshop was held that included representatives from the key stakeholder agencies (Table 1). Although multiple agencies were invited to participate in the workshop, key decisions including selection of criteria, questions to address the criteria, and the final selection of top five zoonotic diseases was made by five pre-selected committee members. The committee members were identified prior to the workshop and included individuals from the Ethiopian Public Health Institute (EPHI), the Ministry of Livestock and Fishery Resources (MoLFR), and the Ethiopian Ministry of Environment and Forestry (MEF).

**Table 1:** The Ethiopia One Health Zoonotic Disease Prioritization Participating Organizations — Addis Ababa, Ethiopia, 2015.

Participating organizations	Abbreviation
Federal Ministry of Health, Ethiopia	FMOH
Ethiopian Public Health Institute	EPHI
Ministry of Livestock and Fishery Resources, Ethiopia	MoLFR
Ministry of Environment and Forestry, Ethiopia	MEF
World Health Organization	WHO
United States Department of Agriculture	USDA
U.S. Centers for Disease Control and Prevention	CDC
Defense Threat Reduction Agency/Cooperative Biological Engagement Program	DTRA/CBEP
The Ohio State University	OSU
Food and Agriculture Organization of the United Nations	FAO
Armauer Hansen Research Institute/Swiss Tropical and Public Health Institute	AHRI/STPHI

### 2.1 Selection of zoonotic diseases for prioritization

The first step of the process was to identify a country-specific list of zoonotic diseases of potential concern. Subject matter experts from the Ministries, as well as local WHO and CDC staff provided expert opinion on the proposed list of diseases for consideration in Ethiopia. EPHI, MoLFR and CDC circulated the list of potential diseases for inclusion and final selection was based on input from subject matters experts and a literature review.

### 2.2 Literature review

Data on the burden of zoonotic diseases in Ethiopia were identified through an extensive literature search. Forty-three zoonotic diseases were included in the literature review: 18 zoonotic diseases were associated with viral infection, 18 with bacterial infection, and 7 with parasitic pathogens. Peer-reviewed literature citing disease incidence, prevalence, morbidity, disability-adjusted life years (DALYs), and mortality were collected. If information for a particular zoonotic disease was not available for Ethiopia, data for other East African countries was used. If regional data was not available, global disease data were used. Over 300 articles were reviewed with disease-specific information for Ethiopia or the East Africa region.

NCBI PubMed was used to conduct the initial search. Information regarding human disease severity (e.g. morbidity, mortality, and DALYs), economic burden in animals, and prevention and control strategies (e.g. local wildlife reservoirs and vaccine availability) was compiled. The search used the country name (Ethiopia), disease name, and one of the following terms: “morbidity,” “mortality,” “DALYs,” “cases,” “animals,” “vaccine,” and “wildlife,” combined using the Boolean operator “AND.”

Authors reviewed references from retrieved articles to identify additional relevant publications for inclusion in the literature review. Non-English articles were excluded. Articles published during 1965–2015 were included. All articles were collated and shared electronically with workshop participants.

In addition to literature found via PubMed, data publicly available on websites of the World Health Organization (WHO), the United Nations Food and Agriculture Organization (FAO), and the Institute for Health Metrics and Evaluation's 2010 Global Burden of Disease (GBD) Survey were also included.

### 2.3 Criteria selection

Through group discussion and consensus, the workshop participants identified five criteria for quantitative ranking of the 43 zoonotic diseases. Once the five criteria were chosen, each member of the selection committee individually indicated their preferences for the relative importance of each criterion to help generate a final group of weights for each criterion. The criteria and weights assigned to each one of them are listed in Appendix A.

### 2.4 Question selection for each criterion

A categorical question for each criterion was selected through group discussion. The questions were designed to address the criteria using data generated from the literature review for each of the 43 zoonotic diseases. The questions had binomial (yes/no) or ordinal multinomial (1–5%, 5–10%, 10–20%, etc.) answers. The ordinal nature is necessary for the scoring process, and was guided by participant preference and the available data.

### 2.5 Disease weighting and final ranking

A decision tree was designed using Microsoft Excel and was used to determine the final disease ranking. Each weighted criterion was applied across all diseases, and scores were assigned based on the response to each question. Data compiled during the literature review were used to determine appropriate responses for each question for all zoonotic diseases under consideration. The scores for all five questions were summed and then normalized such that the highest final score was 1.

Workshop participants reviewed the numerical scores generated and engaged in further discussion to determine the final five prioritized diseases. Finally, the selection committee members voted on the top five zoonotic diseases for Ethiopia.

## 3 Results and discussion

Although this workshop has been conducted in other countries, the criteria selected and the final five prioritized zoonotic diseases are unique to Ethiopia. Criteria selected by the workshop participants are listed in order of importance below. Detailed descriptions can be found in Appendix A.

### 3.1 Severity of human disease in Ethiopia

Diseases having the highest death rate (i.e., number of deaths per population) in humans were deemed to have priority and the criterion was given the highest weight. However, death rate data for each of the 43 zoonotic diseases of concern were not available. A proxy was established in which the diseases were ranked based on their known presence in Ethiopia and the global case-fatality rate (CFR). A disease was given full weight for this criterion if there was any data indicating its presence in Ethiopia and the disease had a high CFR (≥ 5%). The next highest credit (two-thirds) was given for diseases which were known to be present in Ethiopia, but had a low CFR (< 5%). The lowest credit (one third) was given for diseases not present, or not known to be present, in Ethiopia, but with a high CFR (≥ 5%). No credit was given to diseases not present or not known to be present in Ethiopia and with a low CFR (< 5%).

### 3.2 Proportion of human disease attributed to animal exposure

Diseases that are not known to spread from person to person (and thus all cases result from animal exposure) were assigned the full weight of the criterion (e.g. rabies). Diseases which can spread from animal to person and then are maintained from person to person received half credit (e.g. ebola). And finally, diseases known to spread mainly between people (cases rarely originating from animal exposure) received no credit.

### 3.3 Burden of animal disease

Priority was given to diseases that have negative impacts at the household level in Ethiopia by causing disease or production losses in livestock. Assessing the burden of disease in animals was challenging because data were available for very few of the 43 diseases. For those diseases with data available, they differed across regions and species. Diseases were ranked and assigned weights based on whether the disease was present or not present (or not known to be present) in Ethiopia, and whether the disease causes production losses. If the effect on livestock production was unknown, the final weight was assigned based on whether or not the disease was an OIE reportable disease. If the disease is present in Ethiopia and 1) causes production losses or 2) is an OIE reportable disease it received the full weight of the criterion. Diseases present in Ethiopia that 1) do not cause production losses or 2) are not OIE reportable received the next highest credit (two-thirds). Diseases not known to be present in Ethiopia and 1) known to cause production losses or 2) are OIE reportable received the lowest credit (one-third). Diseases not known to be present in Ethiopia and 1) not known to cause production losses or 2) not OIE reportable did not receive credit.

### 3.4 Availability of interventions

A full weight was assigned to diseases for which vaccines targeting animals existed. Half credit was given to diseases that had vaccines or medical intervention available for people, but not an animal vaccine. No credit was assigned when interventions for animals or people was not available.

### 3.5 Existing inter-sectoral collaboration

Finally, the group prioritized diseases in which inter-sectoral collaboration is already present within Ethiopia and these diseases received full credit for this criterion. Half credit was given to diseases with prior or weak collaborations.

Based on the decision tree analysis using these five criteria, the final normalized scores for the 43 diseases under consideration were tabulated (Table 2).

**Table 2:** Raw scores and normalized weights for zoonotic diseases in Ethiopia utilizing the prioritization tool.

Disease	Raw score	Normalized final score
1. Rabies	0.89	1.00
2. Echinococcus	0.73	0.82
3. Anthrax	0.72	0.81
4. Brucellosis	0.65	0.72
5. Leptospirosis	0.65	0.72
6. Q fever (*Coxiella burnetii*)	0.65	0.72
7. Salmonella	0.65	0.72
8. Mycobacterium bovis	0.63	0.71
9. Tularemia (*Francisella tularensis*)	0.58	0.65
10. Leishmania	0.56	0.63
11. Cysticercosis/Taeniasis	0.55	0.62
12. Toxoplasma	0.55	0.62
13. Listeria	0.53	0.60
14. Schistosoma	0.52	0.58
15. Avian Influenza	0.52	0.58
16. Campylobacter	0.48	0.54
17. *E. coli*	0.48	0.54
18. Trypanosoma	0.47	0.53
19. Streptococcus suis	0.44	0.50
20. Rift Valley Fever	0.44	0.49
21. Bartonella	0.44	0.49
22. Japanese Encephalitis	0.43	0.49
23. MRSA (*Staphylococcus aureus*)	0.38	0.42
24. Trichinella	0.37	0.41
25. West Nile Virus	0.36	0.40
26. Eastern Equine Encephalitis Virus	0.35	0.39
27. Hendra Virus	0.35	0.39
28. Yellow Fever Virus	0.35	0.39
29. Ehrlichia	0.30	0.34
30. Lyme disease (*Borrelia burgdorferi*)	0.30	0.34
31. Hanta virus	0.28	0.32
32. Scrub typhus (*Orientia tsutsugamushi*)	0.28	0.32
33. Plague (*Yersinia pestis*)	0.27	0.30
34. Rocky Mountain spotted fever (*Rickettsia rickettsii*)	0.27	0.30
35. MERS-CoV	0.26	0.29
36. Hepatitis E	0.25	0.28
37. Western Equine Encephalitis Virus	0.24	0.27
38. Dengue	0.20	0.23
39. Venezuelan Equine Encephalitis	0.17	0.19
40. Crimean Congo Hemorrhagic Fever virus	0.14	0.16
41. Nipah	0.14	0.16
42. Lassa	0.09	0.10
43. Ebola	0.07	0.08

After further discussion and voting by the selection committee, five zoonotic diseases were selected and ranked from among the top ten diseases for initial intersectoral engagement by human and animal health agencies. The five prioritized diseases were rabies, anthrax, brucellosis, leptospirosis, and echinococcosis (Table 3). The prioritized diseases were selected based on a combination of published information and expert opinion.

**Table 3:** Final disease rankings from the Ethiopian One Health Zoonotic Disease Prioritization workshop, 2015.

Disease	Final ranking
Rabies	1
Anthrax	2
Brucellosis	3
Leptospirosis	4
Echinococcus	5

To facilitate inter-sectoral collaboration and effectively address the impact of the prioritized zoonotic diseases, workshop participants recommended the following next steps: 1) establish a One Health-focused Zoonotic Disease Unit with representation from the animal and human health agencies, 2) develop a national strategy to jointly address the five prioritized zoonotic diseases, which could be one of the primary tasks for the joint Zoonotic Disease Unit, 3) engage leadership across different ministries to support the One Health program platform and assist in coordination of the prioritized zoonotic diseases, 4) strengthen veterinary public health workforce development in collaboration with the Field Epidemiology and Laboratory Training Program, and 5) the prioritized disease list should be reviewed every 2–5 years in order to address new emerging zoonotic disease threats and incorporate knowledge acquired through enhanced surveillance and laboratory diagnostics.

Effective implementation of prevention and control strategies for the prioritized zoonotic diseases requires sustained collaboration among both the animal and human health sectors. To facilitate this, the workshop participants recommended the creation of a One Health-focused Zoonotic Disease Unit, which would include staff from EPHI and MoLFR or other appropriate animal health agencies. The proposed unit would develop a national zoonotic disease strategy and coordinate efforts between the human and animal health sectors to jointly address the selected zoonotic diseases and respond to outbreaks in people and animals. Such a unit would enhance inter-sectoral linkages, facilitate efficient utilization of scarce resources, and capitalize on various sectors' capabilities to improve prevention and control of zoonotic diseases. Similar collaborative units created in other East African countries such as Kenya and Tanzania have helped to advance zoonotic disease prevention and control activities (unpublished data).

The over-arching objective of the zoonotic disease prioritization workshop was to strengthen multi-sectoral collaborations by jointly identifying the top five zoonotic diseases that are most important for human and animal health in Ethiopia. The final outcome was a list of diseases that animal and human health sectors in Ethiopia, international organizations, and other donor agencies can support for strengthening surveillance in humans and animals, enhancing laboratory capacity, developing prevention and control strategies, and conducting joint outbreak investigations. Similar multi-sectoral collaborative efforts have been implemented in other countries [13].

Rabies, brucellosis, and anthrax are vaccine-preventable diseases; however, vaccine interventions should target animals, requiring sustained intersectional collaboration between human and animal health agencies. Appropriate interventions have brought these diseases under control in much of the developed world. These successes can be replicated in many developing countries with appropriate investment in resources. GHSA provides an opportunity to help developing countries control the burden of critical zoonotic diseases that affect human and animal health and also adversely impact the productivity of livestock. Intersectoral collaborative platforms built to address endemic zoonotic diseases will be essential in effectively responding to newly emerging zoonotic diseases.

Limitations of this process included the lack of data available for zoonotic diseases and the subjective, semi-quantitative nature of the criteria selection process. The final disease ranking may have been impacted by the lack of data such that diseases not present in the published literature were not known to be present in Ethiopia and therefore, received lower scores than diseases that are known to be present in the country. The lack of data highlights potential areas for future collaboration and demonstrates the need for enhanced surveillance to improve our knowledge of both the presence and the degree of impact of zoonotic diseases in Ethiopia. Additionally, committee members were identified by the workshop organizers, therefore, there was the potential for selection bias. The prioritized criteria may have been impacted by the input of subject matter experts with strong opinions; however, the workshop fostered collaboration between sectors and encouraged group discussion during the zoonotic disease prioritization process. As such, all stakeholders had opportunity for their opinions to be heard.

## 4 Conclusions

The results of this workshop have been applied to One Health practice in Ethiopia in the following ways: EPHI and the MoLFR have developed an integrated bite case management (IBCM) system to improve rabies surveillance and intersectoral communication; EPHI, MoLFR, CDC and OSU are actively planning a mass canine vaccination campaign, as well as implementing IBCM protocols to improve access to and quality of post exposure prophylaxis for people; EPHI and the MoLFR will be conducting a country-wide brucellosis serosurvey of livestock and people in October 2016; and EPHI and MoLFR are currently developing protocols for increased Anthrax surveillance and diagnostic activities for 2017.

Surveillance and diagnoses of zoonotic diseases requires a One Health approach involving human, animal and environmental sector participation. The One Health Zoonotic Disease Prioritization tool can foster discussion and collaboration between agencies using both qualitative and quantitative methods for analysis of prioritized diseases. Enhancement of public health and veterinary laboratories for the prioritized zoonotic diseases, establishment of joint outbreak response capacity and sharing of surveillance information by animal and human health authorities, and other intersectoral linkages created to tackle the prioritized zoonotic diseases will undoubtedly prepare the country to effectively address newly emerging zoonotic diseases. Intersectoral engagement to establish control and prevention strategies for prioritized zoonotic diseases of greatest importance will reduce and eliminate unnecessary morbidity and mortality in humans and animals and reduce the economic impact of the diseases at the national and household levels while at the same time creating intersectoral linkages and infrastructure improvements needed to rapidly respond to newly emerging health threats.

## Disclosures

The findings and conclusions in this report are those of the authors and do not necessarily represent the views of the Centers for Disease Control and Prevention.

## Funding

This workshop was funded by the Ethiopian Public Health Institute and the Global Health Securities Agenda of the government of the United States of America.

## Conflicts of interest

None.

## Appendix A Criteria selected by the Ethiopian multi-sectoral working group in order of importance

1. Severity of human disease in Ethiopia (criterion weight = 0.23)
  a. Disease present, high (≥ 5%) CFR (score criterion weight = 1)
  b. Disease present, low (< 5%) CFR (score = 0.67)
  c. Disease not known to present, high (≥ 5%) CFR (score = 0.33)
  d. Disease not known to be present, low (< 5%) CFR (score = 0)
2. Proportion of human disease attributable to animal exposure (criterion weight = 0.21)
  a. Sustained animal to human transmission (no human to human) (score = 1)
  b. Human to human transmission possible, but not sustained (score = 0.50)
  c. Human to human sustained transmission (score = 0)
3. Burden of animal disease (criterion weight = 0.20)
  a. Disease present, loss of production yes (score = 1)
  b. Disease present, loss of production unknown, OIE reportable yes (score = 1)
  c. Disease present, loss of production no (score = 0.67)
  d. Disease present, loss of production unknown, OIE reportable no (score = 0.67)
  e. Disease not present, loss of production yes (score = 0.33)
  f. Disease not present, loss of production unknown, OIE reportable yes (score = 0.33)
  g. Disease not present, loss of production no (score = 0)
  h. Disease not present, loss of production unknown, OIE reportable no (score = 0)
4. Availability of interventions (criterion weight = 0.19)
  a. Animal vaccine (score = 1)
  b. Human intervention (vaccine or treatment) (score = 0.50)
  c. Neither (score = 0)
5. Existing inter-sectoral collaboration (criterion weight = 0.17)
  a. Yes, current strong collaboration (score = 1)
  b. Yes, previous or weak collaboration (score = 0.50)
  c. No (score = 0)
